# The hypoglycemic effects of *Juglans regia* L. internal septum in type 2 diabetic patients: A double-blind, randomized, placebo-controlled clinical trial

**DOI:** 10.34172/jcvtr.2023.31835

**Published:** 2023-09-23

**Authors:** Fatemeh Afra, Arman Zargaran, Nooshin Shirzad, Mahboobeh Hemmatabadi, Mahbube Ebrahimpur, Mehrdad Karimi, Mahnaz Khanavi, Mehrzad Mirshekari, Soha Namazi

**Affiliations:** ^1^Clinical Pharmacy Department, Faculty of Pharmacy, Tehran University of Medical Sciences (TUMS), Tehran, Iran; ^2^Department of Traditional Pharmacy, School of Persian Medicine, Tehran University of Medical Sciences, Tehran, Iran; ^3^Endocrinology and Metabolism Research Center (EMRC), Tehran University of Medical Sciences, Vali-Asr Hospital, Imam Khomeini Complex Hospital, Tehran, Iran; ^4^Endocrinology and Metabolism Clinical Sciences Institute, Endocrinology and Metabolism Research Center, Tehran University of Medical Sciences, Tehran, Iran; ^5^Elderly Health Research Center, Endocrinology and Metabolism Population Sciences Institute, Tehran University of Medical Sciences, Tehran, Iran; ^6^Pharmacognosy Department, School of Pharmacy, Tehran University of Medical Sciences, Tehran, Iran; ^7^Research Center for Rational Use of Drugs, Tehran University of Medical Sciences (TUMS), Tehran, Iran

**Keywords:** Diabetes mellitus, *Juglans regia*, Herbal medicine, Insulin resistance, Trial

## Abstract

**Introduction:**

The internal septum of *J.regia* is traditionally used to control diabetes, and its effectiveness has been shown in animal studies. Accordingly, human clinical trials are needed to confirm its effectiveness on hemoglobin A1c (HbA1c), fasting blood sugar (FBS), blood insulin level, and insulin resistance as a complementary for better control of type 2 diabetes.

**Methods:**

This study was a randomized, double-blinded, controlled trial. The lyophilized powder of extract of the internal septum of *J.regia* was used to fill the capsules. Sixty type 2 diabetic patients were randomly divided into two groups. 500 mg capsules three times daily before meal was added to their routine drug regimen, and HbA1c, FBS, and blood insulin level were checked at the baseline and after three months.

**Results:**

Sixty patients completed the study. The mean(±SD) age of patients was 49.1(10.2) and 50.9(12.7) years in the placebo and *J.regia* groups, respectively. We observed that *J.regia* internal septum increases the level of HbA1c by about 0.02 units, but this effect was not significant (MD=0.02,95%CI=-0.36 to 0.40, *P*=0.93). Regarding the impact of capsules on insulin level, it seems that *J.regia*-containing capsules can raise insulin level by one unit. However, it was not significant (MD=1.01,95%CI=-0.86 to 2.88, *P*=0.28). As for FBS, it can cause a decrease of four units, but this effect is also not significant (MD=-3.98,95%CI=-18.33 to 10.37, *P*=0.58).

**Conclusion:**

Based on our study, the internal septum of *J.regia* has no significant effect on HbA1c, FBS, and insulin resistance. Moreover, no specific adverse reaction was observed in any of the patients.

## Introduction

 Diabetes is a chronic disease with the highest economic burden worldwide because of its long-term complications. Furthermore, with the development of communities and the spread of sedentary lifestyles and unhealthy eating habits, the prevalence of type 2 diabetes has increased. Therefore, effective blood glucose control can prevent or delay these complications and improve the quality of life of these patients.^1-3^ Many agents are currently used as injectable and non-injectable forms for controlling blood glucose. Despite being somewhat effective in controlling blood glucose, all these agents’ long-term use can lead to adverse reactions. Some of the complications that can arise include both common and mild ones like hypoglycemia, headache, weight gain, gastrointestinal upset, as well as severe and potentially life-threatening complications such as pancreatitis and heart failure exacerbation.^4,5^ Moreover, despite using multiple anti-diabetic agents, some patients can still not attain optimal blood glucose targets.^6^ Therefore, finding new treatment methods and drugs with fewer side effects for better blood glucose control of diabetic patients is welcomed.

 Recently, there has been an increase in the use of traditional medicine and herbal products for treating diabetes because they are believed to have comparable or superior therapeutic effectiveness with minimal adverse reactions.^7^ Furthermore, numerous conventional remedies for diabetes are sourced from botanical substances. For instance, the first-line treatment of diabetes is metformin, which has its discovery history rooted in a herbal product (*Galega officinalis*), and its potential benefits are still being researched, in addition to lowering blood glucose.^8,9^ Since scientific evidence is necessary to prove the effectiveness of herbal products, So, the effectiveness of these substances were investigated in RCTs.^7,10^

 Also, Herbal products have been used in Iranian traditional medicine to treat diseases. One of these most well-known herbal products in Iranian traditional medicine is the walnut plant with the scientific name “Juglans regia L.” from the Juglandaceae family.^10-12^

*J.regia* has numerous chemical compounds present in its various parts. The leaves contain phenolic acids, tannins, flavonoids, and essential fatty acids, while the fruits have tocopherols, phytosterols, and tannins. Additionally, the green husks contain glucose, citric acid, and malic acid. These are some of the chemical components found in *J.regia*.^13^

*J.regia* is also high in omega-3 unsaturated fatty acids and antioxidants, which can improve gastrointestinal function, lower blood glucose and ultimately reduce the risk of cardiovascular disease. Its beneficial cardiovascular effects include lowering blood cholesterol, increasing the high-density to total cholesterol ratio, reducing inflammation, and improving arterial function.^3,14,15^ The European Society of Cardiology (ESC) 2019 guidelines also mention using walnut to reduce cardiovascular disease.^16^ One of the components of *J.regia*, which has these mentioned properties, is the internal septum. *J.regia* internal septum is rich in polyphenols, flavonoids such as quercetin, gallic acid, protocatechuic acid, dihydrophaseic acid, etc.In Iranian traditional medicine, its decoction is widely used to control diabetes. It is rich in phenolic acids and flavonoids, resulting in its anti-diabetic properties.^17^

 Additionally, various animal studies were conducted to demonstrate its efficacy, including its ability to reduce blood glucose.^18,19^

 Furthermore, it was shown that *J.regia* internal septum has no acute or subacute toxic effects or death in terms of 1000 mg/kg of internal septum extract, and its lethal dose was reported to be more than 5000 mg/kg.^20^

 Considering all the information above, as previously stated, there is a necessity for novel medications that have satisfactory safety and efficacy measures for managing diabetes. Therefore, based on animal studies and the traditional use of the internal septum of *J.regia* to lower hemoglobin A1c (HbA1c), fasting blood sugar (FBS), and insulin resistance, this clinical trial designed to assess the effectiveness of *J.regia* internal septum containing capsules on HbA1c, FBS, blood insulin level, and insulin resistance.

## Materials and Methods

###  Ethical issues

 The study design complied with the guidelines of the Helsinki Declaration (1989 revision). The ID number of IR.TUMS.TIPS.REC.1399.133 was dedicated to the study protocol from the Research Ethics Committee of the Research Institute of Pharmaceutical Sciences, Tehran University of medical sciences (TUMS). Then, this study was registered in the Iranian Registry of Clinical Trials with registration ID: IRCT20201229049877N1. The informed consent form was provided to all patients before the drug/placebo use, and all patients could withdraw from the study at any time.

###  Preparation of the materials

####  Drug preparation

 At first, *J.regia* fruits were collected from Tuyserkan, Hamedan, Iran. Then it was identified by the herbarium center at the Faculty of Pharmacy of TUMS (Scientific name: *Juglans regia* L.; Family: Juglandaceae; Voucher number: PMP-2665).

 Then, to prepare the capsules containing the internal septum of *J.regia*, hydro-alcoholic extract (70% ethanolic extract) was taken from 100 kilograms (Kg) of the dried internal septum by percolation method (48 hours; three times).^21^ The solvent was then evaporated by a rotary evaporator. The obtained extract was dried using a freeze dryer and turned into a dry powder.

 Zero-size capsules were used to make the drug capsules, and 500 milligrams (mg) of the dry extract powder was added to each capsule. The placebo capsules were identical to the drug capsules in appearance. But 500 mg of microcrystalline cellulose was used to fill them instead of the dry extract powder.

 This dose was calculated using the formula to convert animal to human dose.^22^ (Formula 1)

 Formula 1:


$$\begin{array}{l} Human\;equivalent\;dose\;\left(HED\right)\;\left(\frac{mg}{Kg}\right)=\\ Animal\;no\;observed\;adverse\;effect\;level\;\left(NOAEL\right)\left(\frac{mg}{Kg}\right)\times \frac{Animal\;weight\left(Kg\right)}{Human\;weight\left(Kg\right)} \end{array}$$

####  Standardization of extract

 The product was standardized based on the amount of its total polyphenols based on gallic acid by spectrophotometry. Folin Ciocalto (FC) method was used to determine total phenolic content.^23^ At first, 0.1 ml (2.5 mg/dl) of hydroalcoholic extract was added to 7.5 ml of distilled water. Moreover, 0.5 ml FC reagent was added. The resulting solution was stirred for 3 minutes. Then, 1.5 ml (200mg/ml) sodium bicarbonate was added to this solution. Then, this solution was kept for 3 hours in a dark environment at room temperature. So, the sample was analyzed with a UV mini 1240 UV-Visible spectrophotometer (Shimadzu, Japan) at 765 nm. The total flavonoid content of the extract was determined using the aluminum chloride colorimetric method.^24^ For this purpose, 1 ml of the hydroalcoholic extract of *J.regia* internal septum (100 μg/ml) was added to 4 ml of distilled water. It was stirred with 0.3 ml of 5% sodium nitrite in a 10 ml volumetric flask. After 5 minutes, 0.3 ml of 10% aluminum chloride solution and 2 ml of sodium hydroxide (1 mole per liter) were added to the solution, and the volume was brought to a volume of 10 ml with distilled water. Then this solution was kept for 10 minutes in a dark environment at room temperature. So, the sample was analyzed with a UV mini 1240 UV-Visible spectrophotometer at 510 nm. Quercetin solution in 0-100 μg/ml concentrations was used as a standard. The results were expressed as milligrams of quercetin equivalent per gram of dry extract.

###  Clinical phase

####  Study design and outcomes

 This study was designed as a double-blind, randomized, parallel, placebo-controlled clinical trial. Both patients and researchers were blinded to avoid allocation errors. Only the manufacturer knew the difference between drug and placebo batch numbers.

**Figure 1:**
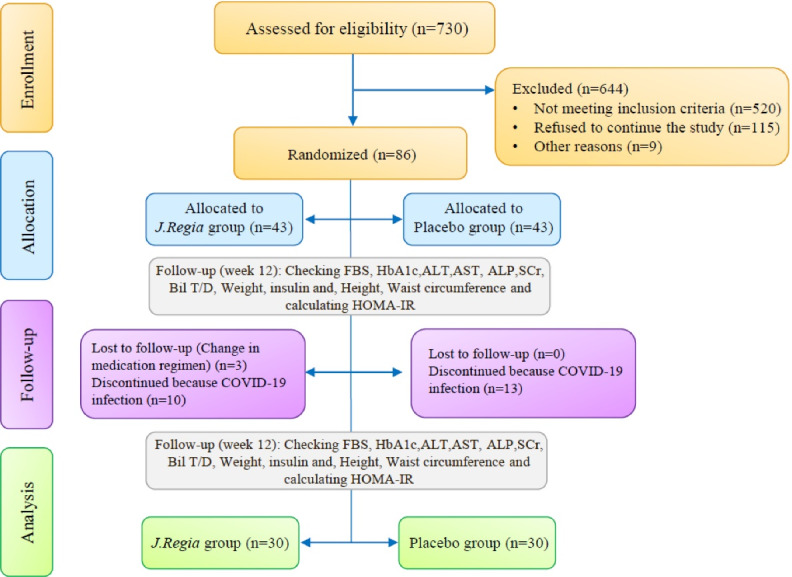
Study design process: FBS = Fasting blood sugar, HbA1c = Hemoglobin A1c, ALT = Alanine transaminase, AST = Aspartate aminotransferase, ALP = Alkaline phosphatase, SCr = Serum creatinine, Bil T/D = Bilirubin total and direct, HOMA-IR = Homeostatic Model Assessment for Insulin Resistance

 Among patients referred to the outpatient diabetes clinic of Imam Khomeini Complex Hospital, Professor Yalda’s diabetes clinic, and Clinic No. 2, diabetes and metabolic diseases affiliated to TUMS, Tehran, Iran, 730 patients were assessed for eligibility. Among them, 644 patients were excluded based on exclusion criteria. Then 86 patients were randomly divided into two groups of 43 patients with a 1:1 ratio to control and intervention groups using the block randomization method. During the follow-up period, 23 patients did not continue the study. Finally, 60 patients were analyzed (Figure 1).

 Lowering HbA1c in patients with type 2 diabetes was considered the main goal. Also, reducing FBS and insulin resistance were considered as the secondary outcomes of this study.

####  Inclusion and exclusion criteria

 Any patient aged 25 to 70 years with HbA1c between 7 and 9% and FBS 130 to 250 mg/dl while taking Metformin and other anti-diabetic agents (non-insulin) were included after obtaining personal consent. Furthermore, the admission of pregnant and lactating patients and those with a history of allergic reactions to *J.regia*, autoimmune and cardiovascular diseases (including ischemic heart disease and/or heart failure with ejection fraction < 45%), uncontrolled thyroid disease (Thyroid-stimulating hormone (TSH) level > 4.5 mIU/L or < 0.4mIU/L), history of diabetic ketoacidosis, renal failure (estimated glomerular filtration rate (eGFR) < 60 ml/min according to Cokroft and Gualt; formula,2), acute hepatitis (alanine transaminase (ALT) and aspartate aminotransferase (AST) > 10-20 fold upper limit of normal), cirrhosis, proliferative retinopathy and severe weight loss (At least 10% during 6 months)^10^ were avoided. Besides, this study did not include patients taking drugs that can raise blood glucose, such as Corticosteroids, Tacrolimus, etc.^25^, and herbal medicines containing *J.regia*.

 Formula 2:


$$eGFR=\frac{140-Age\left(Weight\;in\;Kg\right)\left(0.85\;if\;female\right)}{72*serum\;creatinine}$$

###  Clinical trial

 Patient selection began in November 2020 and continued until October 2021. Drug/Placebo was administered three times daily before meals. Patients were followed for 12 weeks.

 At the beginning and end of the follow-up period (12 weeks), blood samples were drawn to assess the blood levels of HbA1c (measured by Sebia kits, France), FBS (measured by Audit kits, Iran), insulin (using chemiluminescent immunoassay (*CLIA*) method) (measured by Siemens kits, Germaney), AST, ALT, alkaline phosphatase (ALP), total and direct bilirubin, serum creatinine. Moreover, Homeostatic Model Assessment for Insulin Resistance (HOMA-IR)^26^ was calculated to analyze insulin resistance (Formula 3). Blood samples were taken from patients while fasting for 8-12 hours. To prevent assessment bias in the reported values, all patients were referred to a specific laboratory in Tehran. Moreover, the patients’ weight was measured equally by one person in the diabetes clinic where the study was performed. Patients were asked to have a fixed diet and physical activity.

 Formula 3:


$$HOMA-IR=\frac{Glucose\left(\frac{mg}{dl}\right)*insulin\left(\frac{micU}{L}\right)}{405}$$

The patients were contacted in person during the study in weeks 0 and 12. In the meantime, the patients were followed by telephone and social networks. The reports of possible side effects were received from patients and evaluated based on the Naranjo scale. In this way, scores of 9 or 10 are considered as “definitely” adverse drug reaction, and scores of 5-8, 1-4, and less than one are considered as “probable”, “possible”, and “doubtful adverse drug effect, respectively.^27^

 Patients were asked to deliver empty drug/placebo cans to assess their compliance. According to available articles, adherence is considered appropriate if the patient takes more than 80% of the drugs in RCTs.^28^

###  Statistical analysis

 The minimum sample size considering the significance level of 0.05 with a statistical power of 80% to detect at least a standardized effect size of Δ=ε/σ=0.65 ^29^, based on HbA1c with an addition of 10% to cover up the possible dropouts, the sample size was determined as 40 participants in each group.

 Data were analyzed using STATA version 14.2. Descriptive analyses of baseline variables were expressed as mean, standard deviation (SD), median, interquartile range (IQR), number, and percentage as appropriate.

 Analyses of variance/covariance (ANOVA/ANCOVA) were applied to adjust between-group effects for potential confounders, covariates, or baseline measurements.^30^

 The basic variables which had considerable differences between the two studied groups were entered into multivariate analysis for the possibility of covariate effects. They were kept in the final analysis model if they changed the effect size.

**Figure 2:**
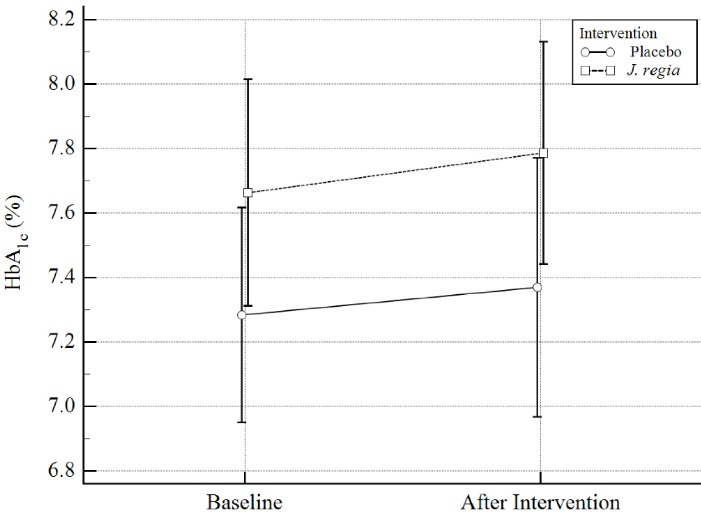
Hemoglobin A1c (HbA1c) distribution (%) in pre & post intervention by study groups. The dispersion index is 2SE

**Figure 3:**
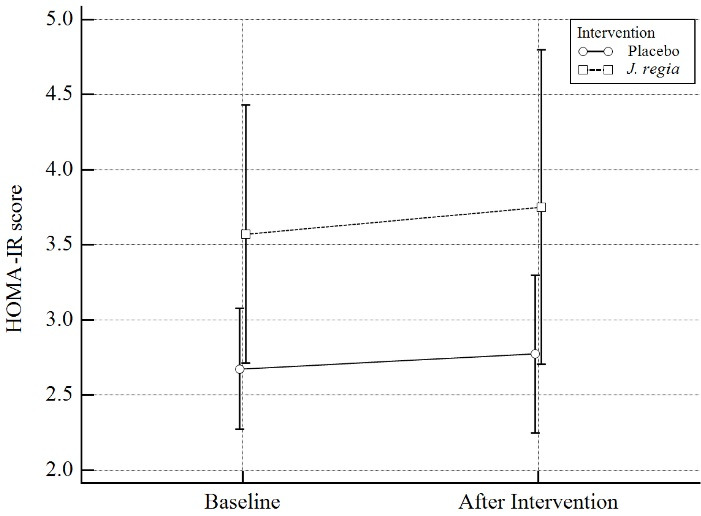
Homeostatic Model Assessment for Insulin Resistance (HOMA-IR) score distribution in pre & post intervention by study groups. The dispersion index is 2SE

**Figure 4:**
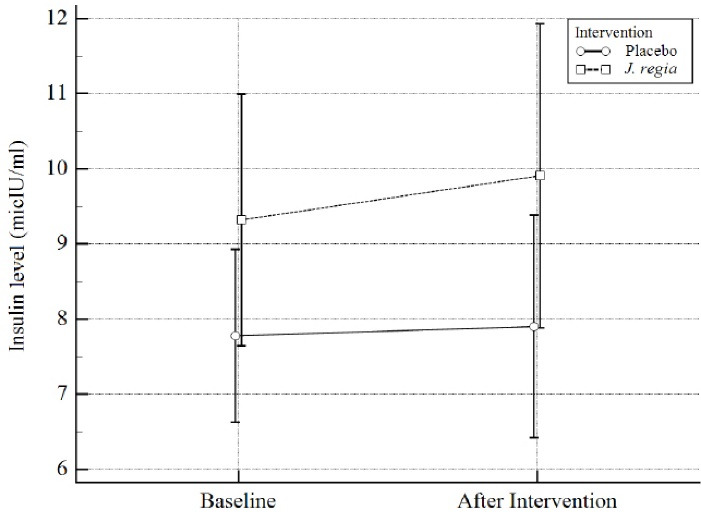
Insulin level distribution by study groups. The dispersion index is 2SE

 Mean difference (MD) and standardized mean differences (Cohen’s d, trivial: SMD < 0.2, small: 0.2 ≤ SMD < 0.5, moderate: 0.5 ≤ SMD < 0.8, large SMD ≥ 0.8)^31^ were calculated for each model as effect size. All *P* values < 0.05 were considered significant. Figures 2 to 4 were designed by MedCalc18.

## Results

###  Drug preparation phase

 The mean (± SD) of the total polyphenolic content of *J.regia* internal septum extract was 74.57 (5.20) mg of gallic acid equivalent per gram of dry extract. The mean (± SD) of the total flavonoid content of these capsules was measured as 14.11 (2.73) mg of quercetin equivalent per gram of dry extract.

###  Clinical phase

 During this study, ten patients from the *J.regia* group and 13 patients from the placebo group were excluded due to COVID-19 infection. Three patients in the drug group were excluded from the study regarding the drug regimen change during the study. Finally, 60 patients completed the study. The compliance of drug use for all 60 patients was 100%. The mean (± SD) age of patients was 49.1 (10.2) and 50.9 (12.7) years in the placebo and *J.regia* groups, respectively. The mean duration [IQR] of their disease was 6 [4-8] and 6 [4-8] years in the placebo and *J.regia* groups, respectively. Patients’ characteristic data were summarized in Table 1, and there was no significant difference between them (*P* > 0.05).

**Table 1:** Characteristic parameters

	**Placebo**	***J.regia***
	**N=30**	**N=30**
Sex, n (%)		
Female	17 (57%)	19 (63%)
Male	13 (43%)	11 (37%)
Age (Year), mean (SD)	49.1 (10.2)	50.9 (12.7)
Disease Duration (Year), median (IQR)	6 (4-8)	6 (4-8)
Weight (Kg), mean (SD)	69.0 (12.5)	73.8 (22.2)
Cr (mg/dl), mean (SD)	0.9 (0.2)	0.8 (0.2)
Adjusted baseline drugs*, median (IQR)	6.0 (3.0-6.0)	7.4 (4.5-8.8)
HbA_1_c (%), mean (SD)	7.3 (0.9)	7.6 (0.9)
FBS (mg/dl), mean (SD)	142.2 (30.7)	148.7 (32.7)
Insulin (micIU/ml), median (IQR)	8.0 (5.1-9.9)	9.0 (5.7-12.8)
HOMA-IR**, median (IQR)	2.5 (1.9-3.4)	3.3 (1.9-4.2)

*Equivalent dose of antidiabetic drugs used by patients (Adjusted based on the effect of anti-diabetic drugs on HbA1c). ** HOMA-IR = Homeostatic Model Assessment for Insulin Resistance.

 After 12 weeks, changes in HbA1c were evaluated as the primary outcome. It was initially seen that the *J.regia* internal septum-containing capsules caused an increase in HbA1c by about 0.4 units. But, when we consider baseline HbA1c as a potential confounding factor, we see this effect as much as a 0.13 increase. However, based on our final model, there were no significant differences in HbA1c between the two groups (Mean differences (MD) = 0.02, 95% CI = -0.36 to 0.40, Standardized mean differences (SMD) = 0.11 (-0.40 to 0.61), *P* = 0.93). Results were summarized in Table 2 and Figure 2.

**Table 2:** HbA_1_c distribution (%) in pre & post intervention by study groups in according to different models

**Model**	**Time point**	***J.regia*** ** (Mean±SE)** **n=30**	**Placebo (Mean±SE)** **n=30**	**Mean Difference** **(95% CI)**	**Cohen’s d ** **(95% CI)**	**Adjusted R**^2^	***P***** value**^$^
Crude	Pre	7.66 ± 0.17	7.28 ± 0.17	-	-	-	-
	Post	7.79 ± 0.18	7.37 ± 0.18	0.42 (-0.10, 0.93)	0.42 (-0.10, 0.93)	0.03	0.11
Adjusted^A^	Post	7.64 ± 0.14	7.51 ± 0.14	0.13 (-0.26, 0.52)	0.18 (-0.33, 0.68)	0.47	0.50
Adjusted^B^	Post	7.60 ± 0.15	7.58 ± 0.15	0.02 (-0.36, 0.40)	0.11 (-0.40, 0.61)	0.55	0.93

$ calculated for intervention based on ANOVA/ANCOVA Models. A: Adjusted for baseline Pre-treatment outcome (calculated based on One-way ANOVA/ANCOVA model). B: Adjusted for baseline Pre-treatment outcome and Potential Covariates (drug history and age) (calculated based on Three-way ANOVA/ANCOVA model).

 Moreover, after 12 weeks, the effects of *J.regia* internal septum-containing capsules on HOMA-IR were studied. In our study, it was observed that *J.regia* internal septum-containing capsules compared with the placebo, do not have a significant effect on the insulin resistance of diabetic patients (MD = 0.15, 95% CI = -0.66 to 0.97, SMD = 0.10 (-0.41 to 0.61), *P* = 0.70). Results were summarized in Table 3 and Figure 3.

**Table 3:** HOMA-IR score distribution in pre & post intervention by study groups in according to different models

**Model**	**Time point**	***J.regia*** ** (Mean±SE)** **n=30**	**Placebo (Mean±SE)** **n=30**	**Mean Difference** **(95% CI)**	**Cohen’s d ** **(95% CI)**	**Adjusted R**^2^	***P***** value**^$^
Crude	Pre	3.57 ± 0.33	2.68 ± 0.32	-	-	-	-
	Post	3.75 ± 0.40	2.77 ± 0.40	0.98 (-0.15, 2.11)	0.45 (-0.07, 0.97)	0.03	0.09
Adjusted^A^	Post	3.34 ± 0.28	3.17 ± 0.28	0.17 (-0.64, 0.98)	0.11 (-0.40, 0.62)	0.54	0.67
Adjusted^B^	Post	3.33 ± 0.28	3.18 ± 0.28	0.15 (-0.66, 0.97)	0.10 (-0.41, 0.61)	0.54	0.70

$ calculated for intervention based on ANOVA/ANCOVA Models. A: Adjusted for baseline Pre-treatment outcome (calculated based on One-way ANOVA/ANCOVA model). B: Adjusted for baseline Pre-treatment outcome and Potential Covariate (disease duration) (calculated based on Two-way ANOVA/ANCOVA model).

 Regarding the effect of *J.regia* internal septum on insulin level, It can raise it by one unit. However, this effect is not statistically significant (MD = 1.01,95% CI = -0.86 to 2.88, SMD = 0.29 (-0.23 to 0.81),* P* = 0.28). Results were summarized in Table 4 and Figure 4.

**Table 4:** Insulin level distribution and FBS distribution by study groups in according to different models

**Outcome**	**Model**	***J.regia*** ** (Mean±SE)** **n=30**	**Placebo (Mean±SE)** **n=30**	**Mean Difference** **(95% CI)**	**Cohen’s d ** **(95% CI)**	**Adjusted R**^2^	***P***** value**^$^
Insulin level (micIU/ml)	Crude	9.91 ± 0.87	7.90 ± 0.86	2.01 (-0.44, 4.45)	0.43 (-0.09, 0.94)	0.03	0.11
	Adjusted^A^	9.38 ± 0.64	8.38 ± 0.64	1.01 (-0.86, 2.88)	0.29 (-0.23, 0.81)	0.54	0.28
FBS (mg/dl)	Crude	147.30 ± 6.17	144.10 ± 6.17	3.20 (-14.28, 20.68)	0.09 (-0.41, 0.60)	-0.01	0.72
	Adjusted^A^	144.09 ± 4.90	148.08 ± 4.99	-3.98 (-18.33, 10.37)	-0.15 (-0.66, 0.36)	0.40	0.58

$ calculated for intervention based on ANOVA/ANCOVA Models. A: Adjusted for baseline Pre-treatment outcome and Potential Covariate (drug history) (calculated based on Two-way ANOVA/ANCOVA model).

 As for FBS, as one of the secondary outcomes, it can cause a decrease of 4 units, but this effect also is not statistically significant (MD = -3.98, 95% CI = -18.33 to 10.37, SMD = -0.15 (-0.66 to 0.36),* P* = 0.58) (Table 4).

###  Adverse effects

 We analyzed probably adverse drug reactions. For this purpose, liver enzymes, kidney function, and serum creatinine were measured before starting and at the end of the study, and no significant difference was observed (*p* > 0.05). Moreover, other adverse drug reactions were evaluated using the Naranjo scale score. Two patients in the placebo group reported the same complication of stomach pain right after taking the capsules, which was calculated as a Naranjo score of 2. Based on the Naranjo score of 1-4, this adverse drug reaction probability is possible (Table 5).

**Table 5:** Naranjo adverse drug reaction probability scale score

**Question**	**Yes**	**No**	**Do Not Know**	**Score of our case **
1. Are there previous conclusive reports on this reaction?	+ 1	0	0	1
2. Did the adverse event appear after the suspected drug was administered?	+ 2	-1	0	0
3. Did the adverse event improve when the drug was discontinued or a specific antagonist was administered?	+ 1	0	0	1
4. Did the adverse event reappear when the drug was re-administered?	+ 2	-1	0	0
5. Are there alternative causes that could on their own have caused the reaction?	-1	+ 2	0	0
6. Did the reaction reappear when a placebo was given?	-1	+ 1	0	0
7. Was the drug detected in blood or other fluids in concentrations known to be toxic?	+ 1	0	0	0
8. Was the reaction more severe when the dose was increased or less severe when the dose was decreased?	+ 1	0	0	0
9. Did the patient have a similar reaction to the same or similar drugs in any previous exposure?	+ 1	0	0	0
10. Was the adverse event confirmed by any objective evidence?	+ 1	0	0	0
		Total Score:2		

Naranjo adverse drug reaction probability scale scores of 9 or 10 indicate that the adverse drug reaction was “definitely”, scores of 5-8 indicate “probable”, scores of 1-4 indicate “possible”; and scores of less than 1 indicate “doubtful.

## Discussion

 The primary aim of this clinical trial was to evaluate the efficacy of *J.regia* internal septum on HbA1c in diabetic patients. Our results showed that capsules containing the internal septum of *J.regia* did not significantly affect HbA1c and insulin secretion, insulin resistance (*P* > 0.05).

 As we mentioned above, after 12 weeks, it was initially seen that the *J.regia* internal septum-containing capsules caused an increase in HbA1c by about 0.4 units. But, when we consider baseline HbA1c as a potential confounding factor, we see this effect as much as a 0.13 increase. It means that a major reason for this increase in HbA1c level is justified by the fact that the baseline level of HbA1c in the *J.regia* group was higher. Furthermore, when we considered the different antidiabetic drugs used as a potential confounding factor, it was observed that this effect is such that about 0.02 increases the level of HbA1c in the *J.regia* group, which is practically considered equal to zero.

 Moreover, in evaluating the effects of *J.regia* internal septum-containing capsules on HOMA-IR, before considering the potential covariates, there is no significant difference between the two groups in HOMA-IR (*P* = 0.09). If we consider the baseline level of insulin resistance, which is 3.57 ± 0.33 in the drug group and 3.75 ± 0.40 in the placebo group, as a covariate, we can still see that there is no significant difference between the two groups (*P* = 0.67). Finally, if we consider the duration of diabetes as a potential covariate, it is observed that it does not affect the final results, and it can be concluded that the duration of diabetes is not a potential covariate in our study.

 Since our study is the first RCT conducted on the efficacy of the internal septum of *J.regia on patients’ FBS, insulin level, and HOMA-IR*, it is impossible to compare our results with other similar articles. However, we compare these results with other clinical trials performed on different parts of the *J.regia*.

 These clinical trials were mostly on the effects of *J.regia* on diabetes, blood pressure, dyslipidemia, weight control, antioxidant and anti-tumor properties, cognitive function, etc. In an RCT on 58 diabetic patients by Hosseini et al twice daily using 200 mg of *J.regia* leaves aqueous extract in two months, significant positive effects were observed to reduce HbA1c (7.6 ± 1.3 mg /dL compared with 8.5 ± 1.6 mg/dL at the baseline, *P* < 0.05), FBS (144 ± 65 mg/dL compared with 165 ± 54mg/dl at the baseline, *P* < 0.05) and increasing insulin level (10 ± 4.7 mg/dL compared with 8.6 ± 4.2 mg/dL at the baseline, *P* < 0.05) compared with the control group.^10^ Abdoli and her colleagues performed an RCT on 37 diabetic patients and showed that using three times daily of 250 mg of *J.regia* leaves aqueous extract can significantly reduce FBS (155.5 ± 28.6 mg/dl compared with 173.1 ± 26.9 mg/dl at the baseline with *P* = 0.002). Postprandial glucose (PPG) (192.8 ± 43.3 mg/dl compared with 240.8 ± 51.7 mg/dl at the baseline with *P* = 0.008) and HbA1c (7.2 ± 0.9% compared with 8.2 ± 0.8% at the baseline with *P* = 0.003) during three months.^32^

 However, in three studies like ours, no such positive significant effects were observed on FBS, HbA1c, and HOMA-IR. An RCT by Ying et al which was performed on 24 type 2 diabetic patients, showed that consuming 56 grams of *J.regia* whole fruit per month for eight weeks cannot significantly reduce HbA1c (-0.0 ± 0.3% in diet with *J.regia* compared with -0.0 ± 0.3% in the diet without *J.regia, P* = 0.85) and insulin resistance (0.2 ± 0.9 in diet with *J. regia* compared with -0.2 ± 0.7 in the diet without *J.regia, P* = 0.10).^33^

 Another study by Rabiei et al observed that using 200 mg per day of *J.regia* hydro-alcoholic leaf extract in 39 diabetic patients did not have significant positive effects on FBS (205 ± 51.9 mg/dl in the placebo group vs. 195.2 ± 38.2 mg/dl in *J.regia* group, *P* = 0.5), PPG (303.2 ± 68.1 mg/dl in placebo group vs. 283.8 ± 45.5 mg/dl in *J.regia* group, *P* = 0.323) and HbA1c (9.8 ± 0.8% in placebo group vs. 9.6 ± 1.1% in *J.regia* group, *P* = 0.421).^29^

 An RCT observed that using 56g per day of whole fruit of *J.regia* in 46 patients for eight weeks had no significant effects on FBS (-0.2 ± 8.8 mg/dl in diet with *J.regia* vs. -1.5 ± 6.8 mg/dl in the control group, *P* = 0.457), fasting insulin level (-0.3 ± 19.6 mIU/ml in diet with *J.regia* vs. -1.7 ± 6.6 mIU/ml in the control group, *p* = 0.626) and HOMA-IR (-0.5 ± 5.7 in diet with *J.regia* vs. -0.3 ± 1.8 in the control group, *P* = 0.81).^34^

 We also measured insulin resistance and did not see a significant decrease in insulin resistance using HOMA-IR.

 There are several reasons for not seeing a significant positive response in our study. These include the following: lack of proper drug absorption after consumption. Patients were asked to take the drug/placebo before their meal, based on previous RCTs of other components of the *J.regia*, including its leaves, as well as to prevent possible food-drug interactions.^10^ However, it is necessary to conduct kinetic studies to understand that this product may need to be prescribed with or without food for better absorption. Another hypothesis is since in Iranian medicine, the decoction of this product was used in liquid form, while these capsules were filled with the dry powder of this extract, which is created by the freeze-drying process that may lead to insufficient dissolution which follows the product undergo a further metabolic process, and delay in absorption of the product. The longer absorption process causes the product to be excreted from the body before complete absorption. And therefore, the effective serum level is not achieved. Moreover, the drug preparation process hypothesizes that the active ingredient which causes anti-diabetic effects may be degraded during the lyophilization of the extract process.^35^ Furthermore, regarding different chemical compounds present in the leaves, internal septum, and whole fruit of *J.regia*, it may be hypothesized that the amount of flavonoids, such as quercetin, which causes antioxidant and antidiabetic effects in *J.regia* leaves, are lower in whole fruit and internal septum which has prevented this effect being observed in whole fruit studies like us.^13^ Another hypothesis raised here is the daily dose of *J.regia* internal septum. In our study, the patients took 1500 mg of capsules containing *J.regia* internal septum lyophilized extract daily. This dose was obtained based on extrapolation of animal studies, as mentioned in the method section.^22^ It raises the possibility that in order to observe the positive effects, a higher dose of the product may be needed. (Considering the toxic dose of the internal septum, it is suggested to increase the total daily dose of drugs in the future study).

 Another possible reason is the duration of consumption of the product. Although according to previous studies^10,32^ and various guidelines, including the American diabetes association (ADA) 2022,^36^ the appropriate follow-up period is three months in terms of the changes in HbA1c. However, this time may be short for herbal medicines and requires a longer period to study their effectiveness.

 Another possible hypothesis is the possible interactions of this product with the other drugs used by the patient, for which no data is available, and further studies are needed to prove this hypothesis.

 However, in view of the above and the review of studies conducted in this field, it is suggested that several other clinical trials with longer duration and higher doses should be performed to evaluate the anti-diabetic properties of *J.regia* internal septum. Our study had two limitations. Firstly, we did not perform pharmacokinetic studies on the product. Secondly, due to COVID-19, there was low attendance of diabetic patients at clinics which was another limitation.

## Conclusion

 It was observed that 500 mg *J.regia* internal septum capsule three times daily has no effects on HbA1c, FBS, and Insulin resistance in the patients with type 2 diabetes. It was observed that it has no side effects. Therefore based on our results, *J.regia* internal septum capsules should not be used as an antidiabetic drug, unless its antidiabetic effect is confirmed in other large clinical trials.

## Acknowledgments

 Authors would like to thank all patients accompanied in this study and also, diabetes clinics of Imam Khomeini Complex Hospital, Professor Yalda and Clinic No. 2 affiliated to TUMS, Tehran, Iran.

## Competing Interests

 The author(s) declared no potential conflicts of interest with respect to the research, authorship, and/or publication of this article.

## Ethical Approval

 The protocol of this RCT was approved by the Research Ethics Committee of the Research Institute of Pharmaceutical Sciences, Tehran University of medical sciences (TUMS). Ethical ID number: IR.TUMS.TIPS.REC.1399.133; Approval date: 2020-12-22

## Funding

 This study was financially supported by Tehran University of Medical Sciences and the Research Center for Rational Use of Drugs, Tehran University of Medical Sciences (TUMS), Tehran, Iran.
